# Increased Expression of RUNX1 in Liver Correlates with NASH Activity Score in Patients with Non-Alcoholic Steatohepatitis (NASH)

**DOI:** 10.3390/cells8101277

**Published:** 2019-10-18

**Authors:** Savneet Kaur, Preety Rawal, Hamda Siddiqui, Sumati Rohilla, Shvetank Sharma, Dinesh M Tripathi, Sukriti Baweja, Mohsin Hassan, Sebastian Vlaic, Reinhard Guthke, Maria Thomas, Rania Dayoub, Chaggan Bihari, Shiv K. Sarin, Thomas S. Weiss

**Affiliations:** 1 Institute of Liver and Biliary Sciences, New Delhi-110070, India; hamda101@yahoo.com (H.S.); shvetanks@gmail.com (S.S.); dineshmanitripathi@gmail.com (D.M.T.); sukritibiochem@gmail.com (S.B.); ol.mohsin@gmail.com (M.H.); drcbsharma@gmail.com (C.B.); shivsarin@gmail.com (S.K.S.); 2 Department of Biotechnology, Gautam Buddha University, Greater Noida-201308, India; rawal.prets86@gmail.com (P.R.); sumatir101@gmail.com (S.R.); 3 Leibniz Institute for Natural Product Research and Infection Biology—Hans Knöll-Institute, 07745 Jena, Germany; bastivl@gmail.com (S.V.); gr.guthke@gmx.de (R.G.); 4 Dr. Margarete Fischer-Bosch Institute of Clinical Pharmacology, Stuttgart, and University of Tuebingen, 72076 Tuebingen, Germany; thomasmasha@gmail.com; 5 University Children Hospital (KUNO), University Hospital of Regensburg, 93053 Regensburg, Germany; Rania.Dayoub@ukr.de

**Keywords:** angiogenesis, inflammation, non-alcoholic fatty liver disease, fatty liver, steatosis, RUNX1

## Abstract

Given the important role of angiogenesis in liver pathology, the current study investigated the role of Runt-related transcription factor 1 (RUNX1), a regulator of developmental angiogenesis, in the pathogenesis of non-alcoholic steatohepatitis (NASH). Quantitative RT-PCRs and a transcription factor analysis of angiogenesis-associated differentially expressed genes in liver tissues of healthy controls, patients with steatosis and NASH, indicated a potential role of *RUNX1* in NASH. The gene expression of *RUNX1* was correlated with histopathological attributes of patients. The protein expression of RUNX1 in liver was studied by immunohistochemistry. To explore the underlying mechanisms, in vitro studies using *RUNX1* siRNA and overexpression plasmids were performed in endothelial cells (ECs). RUNX1 expression was significantly correlated with inflammation, fibrosis and NASH activity score in NASH patients. Its expression was conspicuous in liver non-parenchymal cells. In vitro, factors from steatotic hepatocytes and/or VEGF or TGF-β significantly induced the expression of *RUNX1* in ECs. *RUNX1* regulated the expression of angiogenic and adhesion molecules in ECs, including CCL2, PECAM1 and VCAM1, which was shown by silencing or over-expression of *RUNX1*. Furthermore, *RUNX1* increased the angiogenic activity of ECs. This study reports that steatosis-induced *RUNX1* augmented the expression of adhesion and angiogenic molecules and properties in ECs and may be involved in enhancing inflammation and disease severity in NASH.

## 1. Introduction

Non-alcoholic fatty liver disease (NAFLD) includes a wide compass of liver pathologies, ranging from simple steatosis, usually a mild, benign and non-progressive condition, to non-alcoholic steatohepatitis (NASH), which may progress to liver cirrhosis and ultimately hepatocellular carcinoma (HCC). With NAFLD affecting both children and adults alike, it is postulated to emerge as the leading cause of end-stage liver diseases in the coming years [1]. Several cellular and molecular events conspire and collaborate to transform simple steatosis to NASH to HCC. However, the underlying precise mechanisms of disease pathogenesis and NAFLD progression have just begun to be understood. Some of the newly emerging concepts include iron overload, inflammation, dysregulated fat metabolism, oxidative stress, gut microbiota and angiogenesis [2].

Angiogenesis or new blood vessel formation is a crucial aspect of inflammation and a critical step in tissue damage, healing, and vascular remodeling. Changes in liver vascular architecture have been linked to the progression of fibrosis, cirrhosis and HCC in chronic liver diseases (CLD) [3,4]. Almost all experimental and clinical conditions of CLD, including NASH, have been associated with an over-expression of pro-angiogenic cytokines and related receptors. Although various studies have reported an upregulation of angiogenic factors, particularly VEGF in NASH, the underlying mechanisms that regulate angiogenesis, inflammation and fibrogenesis in NASH pathology remain obscure [5,6,7].

In the current study, we explored the role of Runt-related transcription factor 1 (*RUNX1*) in the pathogenesis of NASH. *RUNX1*, also known as acute myeloid leukemia 1 (*AML1*), is a powerful and pivotal regulator of hematopoiesis and angiogenesis [8,9]. A defect in *RUNX1* is associated with impairment in angiogenesis accompanied by the absence of hematopoietic stem cells [10]. Given the significance of *RUNX1* in angiogenesis and its rarely identified role in NASH, we investigated the expression and function of RUNX1 in NASH pathology by addressing its emergence in endothelial cells (ECs).

## 2. Materials and Methods

### 2.1. Study Subjects and Collection of Samples

Human liver tissues were histologically examined for patients without NAFLD (*n* = 33), patients with simple liver steatosis (*n* = 46) and patients with NASH (*n* = 43) as described earlier [11,12] (for tissue characteristics see Supplementary Table S1). A subset of these samples was used for a mRNA microarray analysis: patients without NAFLD (*n* = 7), patients with simple liver steatosis (*n* = 7), and with non-alcoholic steatohepatitis (NASH) (*n* = 7). The experimental procedures were performed according to the guidelines of the charitable state-controlled foundation HTCR (Human Tissue and Cell Research, Regensburg, Germany), with written informed consent from patients. The study in Germany and the consent form were approved by the local ethical committee of the University of Regensburg (ethics statement 12-101-0048, University of Regensburg, Germany). Additionally, immunohistochemistry (IHC) studies were conducted on liver biopsies collected from NASH patients’ samples (*n* = 16) and control liver tissues (n =10) collected in ILBS, New Delhi (for patient characteristics see Supplementary Table S2). The study performed in India was duly approved by the Human ethics committee of ILBS, New Delhi (ethics approval F25/5/64/ILBS/AC2014/1484). All experiments involving human tissues and cells were carried out in accordance to The Code of Ethics of the World Medical Association (Declaration of Helsinki).

### 2.2. Differential Gene Expression Studies and qRT-PCRs

About 17 differentially expressed genes (DEGs) obtained from a microarray experiment and associated with gene ontology (GO) term angiogenesis were selected for further Taqman quantitative real time-PCR (qRT-PCR) validation studies (Supplementary Table S3A) using a larger cohort of NAFLD liver tissue samples (Supplementary Table S1). For in vitro assays, SYBR Green PCR master mix (Applied Biosystems, Foster City, CA, USA) based qRT-PCR studies were done (Supplementary Table S3B).

### 2.3. Immunohistochemistry Analysis

Samples of human liver tissues were fixed and stained as per standard protocols. IHC scoring was done on a scale of 1–4 by counting RUNX1 positive cells per field. Details of the protocols and antibodies used are given in the supplementary material and Supplementary Table S4.

### 2.4. Culture of Endothelial Cells with Conditioned Medium from Hepatoma Cells Treated with Palmitic Acid

Huh7 cells or mouse primary hepatocytes were treated with 200 µM palmitic acid-BSA (PA) for 48 h according to previously published methods [13]. BSA treated cells served as controls. To investigate the effect of steatotic liver cells on gene expression, human umbilical vein endothelial cells (HUVECs) or LSECs (mouse) were incubated with conditioned medium (CM) from BSA/PA treated Huh7 cells or primary hepatocytes, respectively, for 24 h and then assayed for gene expression. For validation of VEGF in the induction of *RUNX1* gene expression, studies were also conducted by adding VEGF blocking antibody in HUVECs along with CM from BSA/PA treated Huh7 cells.

### 2.5. Induction of RUNX1 Expression in HUVECs 

To study induction of *RUNX1* expression in HUVECs, HUVECs were treated with or without 10 ng/mL VEGF (Himedia Laboratories, Mumbai, India) or TGF-β for 24 h. After 24 h, cells were trypsinized for analysis of RUNX1 gene expression in unstimulated and stimulated cells.

### 2.6. RUNX1 Inhibition and Overexpression in HUVECs 

To elucidate the role of *RUNX1* in inflammation and angiogenesis, steatotic or activated HUVECs were transfected with 50 nmol/mL × 10^6^ cells of either negative control (NC) siRNA (Catalogue no# AM4635) or pre-designed *RUNX1*-specific siRNA (siRNA ID: s229352) (Thermofisher Scientific, Waltham, MA, USA) using lipofectamine 2000 (Invitrogen, Carlsbad, CA, USA) according to the manufacturer’s instructions. Forty-eight hours after transfection, cells were analyzed by qRT-PCR to confirm the knockdown of *RUNX1*. For overexpression studies, HUVECs were transfected with 2μg/L × 10^6^ cells of control plasmid (pControl, empty vector) or RUNX1 plasmid (pcDNA3.1+ /C-(K)-DYK vector with RUNX1b, Gene Script # OHu26354), referred to as pRUNX1, in the absence or presence of 10 ng/mL VEGF. For transfection, lipofectamine 2000 (Invitrogen, Carlsbad, CA, USA) was used according to the manufacturer’s instructions. pcDNA3-EGFP plasmid vector (kind gift from Dr. Vijay) was used as the control of transfection efficiency and expression in all the transfection experiments. Forty-eight hours after plasmid transfection, cells were analyzed by qRT-PCR. HUVECs with loss of RUNX1 or gain of RUNX1 expression were assayed for gene expression of adhesion molecules, angiogenic markers by qRT-PCR, flow cytometry and angiogenic functions by matrigel assays. CCL2 levels were assayed by ELISA in HUVECs under different conditions.

### 2.7. Flow Cytometry Analysis

The labeled antibodies used for flow cytometry are given in Supplementary Table S4. After antibody incubation in PBS for 45 min at 4 °C, the cells were fixed with paraformaldehyde in PBS. Multicolor flow cytometry was performed using FACS Verse (BD Biosciences, San Jose, CA, USA) and minimum of 1 million events using live cells were acquired. Analysis of flow cytometry data was performed using Flow-Jo v10 software (BD Biosciences, San Jose, CA, USA). Unstained cells without any antibody were used as negative controls.

### 2.8. Statistical Analysis

PCR data obtained from patient samples were evaluated for normality distribution by a Shapiro–Wilk test. Statistical differences between the two groups were analyzed by a two-tailed Mann–Whitney U Test or a Student's unpaired *t*-test (in vitro and flow cytometry experiments) and statistical differences between several groups (data from human samples) by a Kruskal–Wallis Test (SPSS Statistics program, IBM, Leibniz Rechenzentrum, München, Germany). A value of *p* < 0.05 was regarded as significant. The Pearson correlation (r) was calculated using the IBM SPSS Statistics program. Each experiment was performed in at least triplicates and data were expressed as means ± SD (standard deviation).

More details and description of additional methods are summarized in Supplementary Files.

## 3. Results

### 3.1. Expression of Transcription Factor RUNX1 is Increased in NAFLD Controlling Differentially Expressed Genes (Degs) Associated with Angiogenesis

In a preliminary microarray study, we compared mRNA expression in either groups of control (N or normal liver), steatosis (S) and NASH (SH) and identified differentially expressed genes (DEGs) followed by a gene enrichment analysis to identify GO-categories. A subset of 17 DEGs associated with GO-terms angiogenesis and in part with hypoxia and lipid metabolism was selected (Supplementary Table S5), and together with known angiogenic genes (*VEGFA*, *FLT1/VEGFR1*, *KDR/VEGFR2*, *CXCR4*, *PPARγ*) [3] further analyzed in a validation study using qRT-PCR. By comparison of the relative mRNA expression of the three groups, N, S and SH, we could confirm a significant differential expression of several genes, but not all in a larger cohort of liver tissue samples (Table 1 and Supplementary Table S6). Among the confirmed DEGs, we found transcription factor *RUNX1*, quite recently described to regulate stellate cell activation in NASH [14], and known targets of RUNX1 comprised of *CCL2, NOS3 (eNOS), PI3KCA* and *PRKCE*. Intriguingly, among the genes, which were not significantly different among the controls and patient groups, were typical angiogenic genes *VEGFA, VEGFR1* and *VEGFR2* (Supplementary Table S6).

### 3.2. RUNX1 Expression Correlates with the Severity of NAFLD

**Figure 1:**
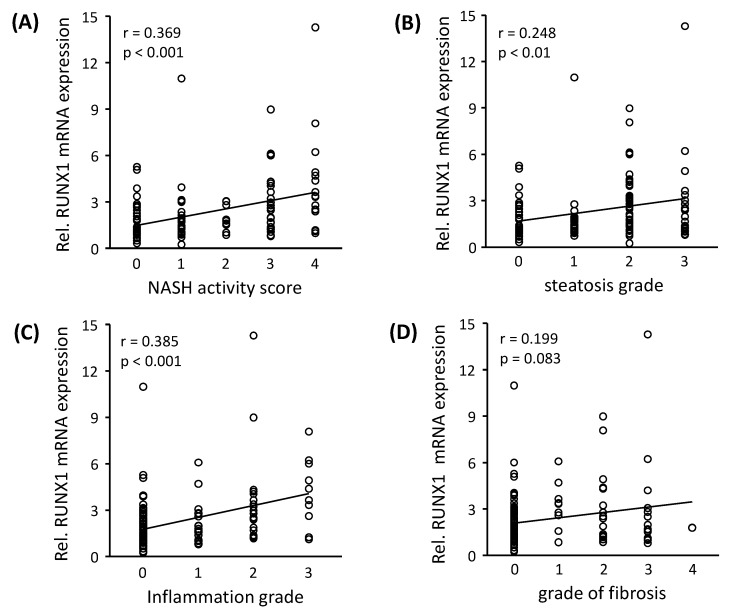
Correlation between *RUNX1* mRNA expression and histopathological parameters. Expression of *RUNX1* mRNA was analyzed by qRT-PCR in liver tissue samples from patients with NASH (*n* = 43), hepatic steatosis (*n* = 46) and normal liver tissue (*n* = 33) and correlated to histopathologic proven (**A**) NASH activity score (**B**) steatosis grade (**C**) inflammation grade and (**D**) fibrosis grade. *HPRT* mRNA expression was determined for normalization, statistical differences between several grades were analyzed by Kruskal-Wallis Test (*p* < 0.05 was considered significant) and ‘r’ denotes the Pearson’s correlation coefficient.

The transcription of *RUNX1* gene is regulated by differential splicing and promoter utilization, which results in three major isoforms: *RUNX1a, RUNX1b* and *RUNX1c* [15]. In all the NAFLD samples analyzed in this study, we found *RUNX1b* as the predominant isoform (data not shown) and therefore, total *RUNX1* expression levels correspond to *RUNX1b*. To analyze the relevance of *RUNX1* mRNA expression with regard to disease progress in our patients, its correlation analysis was performed with the histopathological NASH activity score (NAS), the grade of steatosis, as well as inflammation, and the fibrosis score. *RUNX1* mRNA expression demonstrated a significant positive correlation with NAS, steatosis and inflammation degree (Figure 1A–D). In addition to *RUNX1*, other validated DEGs (Table 1) showing significant correlations with histopathological disease progress are shown in Supplementary Figure S1A–D. RUNX1 target genes, *CCL2* and *PIK3CA* significantly correlated to NAS, steatosis, inflammation grade and fibrosis score, *NOS3* to NAS and inflammation grade and *PRKCE* to NAS and steatosis grade.

**Figure 2:**
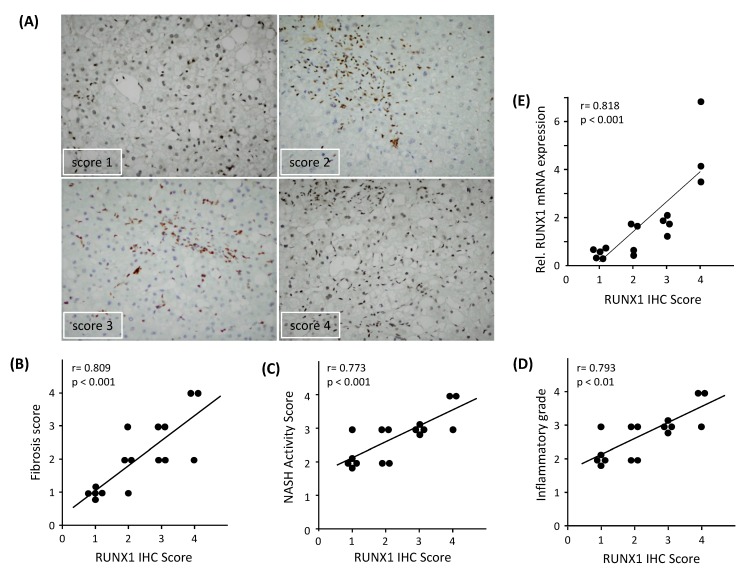
Immunohistochemical (IHC) analysis of RUNX1 expression in NASH patients. (**A**) RUNX1 immunostained images (20× objective) with an increasing number of brown nuclear immuno-positive cells (score 1–4). RUNX1 positivity was mostly observed in the non-parenchymal cells. Hematoxylin stained nuclei were distinguishable from RUNX1-positive brown nuclei. (**B**) Correlation between RUNX1 IHC and NASH activity score (*n* = 16), (**C**) RUNX1 IHC score and fibrosis grade (*n* = 16), and (**D**) RUNX1 IHC score and inflammatory grade (*n* = 16) in NASH patients. (**E**) Correlation between *RUNX1* mRNA and its IHC score in liver tissues of patients (n = 16). The Pearson correlation (r) and statistical significance (*p*) were calculated.

To study RUNX1 protein expression, we performed an IHC analysis of RUNX1 in NASH liver biopsies. NASH patients showed varying degrees of RUNX1 protein expression in the liver (Figure 2A). RUNX1 nuclear positivity was almost absent in parenchymal cells and well evident in liver NPCs, presumably among others in ECs and hepatic stellate cells. Negative antibody controls (with RUNX1 antibody and without secondary antibody) are shown in Supplementary Figure S2. Furthermore, RUNX1 expression was significantly positively correlated with fibrosis score (r = 0.80, *p* < 0.001), NAS (r = 0.77, *p* < 0.001) and inflammatory grade (r = 0.79, *p* < 0.01) in NASH liver tissues (Figure 2B–D) and therefore correlates with NASH disease severity. In addition, RUNX1 protein expression (IHC scores) in the liver tissues of NASH patients were significantly positively correlated with its liver mRNA expression (r = 0.81, *p* < 0.001, Figure 2E).

### 3.3. Palmitic Acid Treated Huh7 Cells Release VEGF and TGF-β-Inducing RUNX1 Gene Expression in ECs

**Figure 3:**
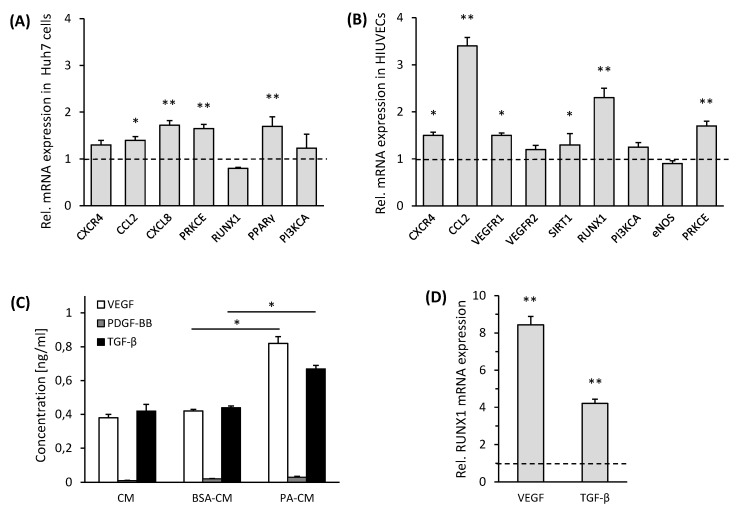
*RUNX1* and angiogenic gene expression in endothelial cells maintained in conditioned medium (CM) from palmitic acid (PA) treated hepatoma (Huh7) cells. (**A**) Huh7 cells treated with 0.2 mM PA for 48 h were analyzed for mRNA expression of *RUNX1* and genes identified as angiogenesis associated DEGs in human NASH samples. The dotted line represents the control showing gene expression in Huh7 cells treated with BSA (*n* = 4). (**B**) HUVECs incubated with CM from PA-Huh7 cells were analyzed for mRNA expression of *RUNX1*, its target and angiogenic genes. The dotted line represents control, showing gene expression in HUVECs treated with CM from BSA-Huh7 cells (*n* = 4). 18S RNA expression was used for normalization. (**C**) Huh7 cells were treated with BSA or PA or CM alone and analyzed for the release of VEGF, PDGF-BB and TGF-β (pg/mL) (*n* = 3). (**D**) Relative *RUNX1* mRNA expression in HUVECs treated with VEGF and TGF-β (10 ng/mL each) for 24 h. Un-induced cells without any manipulation were used as respective controls (Dotted line) (*n* = 3). 18S RNA expression was used for normalization. Data represent mean ± SD. * *p* < 0.05; ** *p* < 0.001.

To investigate if steatosis induces *RUNX1* expression, studies were further carried out in in vitro models of NAFLD. PA treatment of Huh7 cells led to significant increases of lipid droplets in these cells visualized by BODIPY staining (Supplementary Figure S3A). Analysis of mRNA expression of BSA treated control cells (BSA-Huh7) and Huh7 cells treated with PA (PA-Huh7) revealed a low but significant increase in the expression of *CCL2, CXCL8, PPARγ* and *PRKCE *(Figure 3A), of which the latter was also significantly enhanced in steatotic compared to normal human liver samples (Table 1). Of note, *RUNX1* expression was not altered by PA treatment in Huh7 cells. Therefore, in addition to the expression of some angiogenic genes in steatotic hepatocytes, other hepatic cells may contribute to a greater extent to angiogenic gene expression in NASH tissue.

Moreover, HUVECs maintained for 24 h in culture medium (CM) from Huh7 cells treated with PA or BSA demonstrated a substantial upregulation of *RUNX1* and its target gene *CCL2*, as well as the angiogenic gene expression of *CXCR4, VCAM1, VEGFR1* and *PRKCE* after treatment with CM from PA-Huh7 cells (Figure 3B). To verify the impact of CM from PA-Huh7 cells on *RUNX1* expression, HUVECs were solely incubated with 200 µM PA, showing a moderate increase of *RUNX1* mRNA expression compared to the control cells, but considerably less than in PA-Huh7 CM treated HUVECs (Supplementary Figure S3B). However, only the PA treatment of HUVECs resulted in enhanced cell death and hence, this setup was not used in further assays (data not shown). Furthermore, we confirmed our results in mouse LSECs incubated with CM from primary mouse hepatocytes treated with PA for 24 h, showing a more than two-fold increase in the expression of *RUNX1* mRNA compared to control cells (Supplementary Figure S3C).

Then, we analyzed the CMs of PA treated hepatoma cells for angiogenic factors, potentially regulating *RUNX1* expression, such as VEGF, PDGF-BB and TGF-β, well known for their proangiogenic and fibrogenic role [16]. Out of the three factors analyzed, both VEGF and TGF-β were significantly enhanced in the PA-treated CM of hepatoma cells in comparison to BSA-treated conditioned media after 24 h of incubation (Figure 3D). However, levels of PDGF-BB were barely detected (Figure 3C). Next, we studied the effect of both VEGF and TGF-β on the gene expression of *RUNX1* in HUVECs. At similar concentrations (10 ng/mL), both VEGF and TGF-β substantially upregulated the gene expression of *RUNX1* in the HUVECs as compared to the respective non-induced control cells (Figure 3D). Treatment with VEGF antibody reduced the expression of *RUNX1* mRNA by about 60% in HUVECs incubated with CM from PA-Huh7 cells in comparison to that seen in untreated cells under the same conditions (Supplementary Figure S3D). VEGF was used as one of the positive controls and a stimulator of RUNX1 expression in further studies. In conclusion, *RUNX1* expression is not induced in hepatoma cells by PA treatment, but in ECs, mainly through VEGF and TGF-β, released from hepatoma cells after PA treatment.

### 3.4. RUNX1 Enhances Expression of Angiogenic Markers and Adhesion Molecules in HUVECs

**Figure 4:**
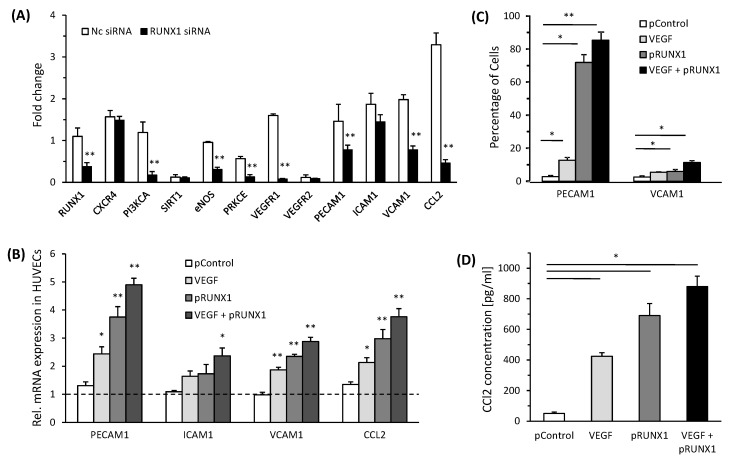
RUNX1 alters expression of angiogenic and adhesion molecules in endothelial cells (**A**) HUVECs, treated with *RUNX1* siRNA or NC siRNA and incubated with CM from PA-Huh7 cells, were analyzed for mRNA expression (fold change) of angiogenic, adhesion molecule and RUNX1 target genes (*n* = 3). (**B**) HUVECs transfected with RUNX1 expression plasmid (pRUNX1), control plasmid (pControl, i.e., empty vector) and/or incubated with VEGF (10 ng/mL) were analyzed for mRNA expression of adhesion molecule and chemotactic genes. HUVECs without any treatment were used as respective controls (Dotted line) (*n* = 3). 18S RNA expression was used for normalization. (**C**) Quantitative analysis of flow cytometry from (**C**) is shown (*n* = 3). (**D**) CCL2 levels (pg/mL) in culture media of HUVECs transfected with RUNX1 expression plasmid (pRUNX1), control plasmid (pControl, empty vector) and/or incubated with VEGF (10 ng/mL) (*n* = 4). Data represent mean ± SD. * *p* < 0.05 and ** *p* < 0.001.

To study the effects of RUNX1 expression on endothelial cell phenotype, we performed both RUNX1 knock-down and overexpression studies in HUVECs. For the RUNX1 knock-down, we attenuated *RUNX1* gene expression in HUVECs treated with CM from PA-Huh7 through *RUNX1* siRNA, which led to a more than 50% reduction in *RUNX1* mRNA expression in HUVECs compared with NC siRNA (Figure 4A, Supplementary Figure S4A) (*RUNX1* siRNA1 is further described as *RUNX1* siRNA). In comparison to controls, RUNX1 knockdown conditions led to a significant decrease in the expression of *VEGFR1* and *RUNX1* target genes, including *PRKCE* and *PI3KCA* in HUVECs cultured with CM from PA-Huh7 (Figure 4A). Hypothesizing the role of RUNX1 in endothelial cell mediated inflammation and leukocyte infiltration, we also evaluated the expression of adhesion molecules and chemotactic factor CCL2 (target gene of RUNX1) in HUVECs. When HUVECs were treated with *RUNX1* siRNA, expression of *VCAM1*, *PECAM1* and *CCL2* was significantly downregulated as compared to that observed in the controls (Figure 4A). On the other hand, transfection with expression vector for RUNX1 (pRUNX1) led to a more than four-fold increase in the expression of *RUNX1* gene in the HUVECs compared to control vector transfected cells (Supplementary Figure S4B). Furthermore, in HUVECs transfected with pRUNX1, mRNA expression of *PECAM1*, *VCAM1* and *CCL2* was markedly higher as compared to that in the controls (Figure 4B) and this was not enhanced by additional VEGF treatment, except for ICAM1 expression. Although we did not study the underlying mechanisms that lead to increased expression of these molecules by VEGF and RUNX1, an additive effect observed after treatment with both VEGF + pRUNX1 suggests that VEGF may be acting through both RUNX1-dependent and -independent mechanisms in inducing the expression of these molecules in endothelial cells. In addition, these results were confirmed by protein expression analysis using flow cytometry for PECAM1 and VCAM1 (Figure 4C, Supplementary Figure S5) as well as ELISA assay for CCL2 (Figure 4D). Thus, RUNX1 can regulate the expression of adhesion molecules and chemotactic factors, such as CCL2, in ECs.

### 3.5. RUNX1 Increases Angiogenic Activity of HUVECs

**Figure 5:**
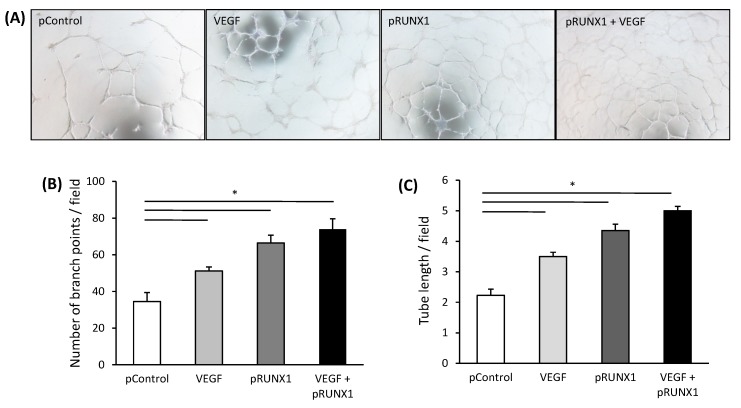
RUNX1 enhances the angiogenic activitiy of endothelial cells. (**A**) Representative tube formation images of HUVECs on matrigel (4× objective) transfected with RUNX1 expression plasmid (pRUNX1), control plasmid (pControl, empty vector) and/or incubated with VEGF (10 ng/mL). (**B**) Average number of branch points per field and (**C**) tube length per field formed by HUVECs on matrigel under conditions described in (**A**) (*n* = 3). Data represent mean ± SD. * *p* < 0.05.

Next, we analyzed if the angiogenic ability of HUVECs was also governed by RUNX1. HUVECs treated with VEGF and/or transfected with pRUNX1 had a substantially augmented angiogenic ability compared to HUVECs transfected with control plasmid, in terms of increased branch points and tube length (Figure 5A–C). The matrigel tube formation ability of HUVECs was also enhanced when cultured in CM from PA-Huh7 cells as compared to that when treated with CM from BSA-Huh7 cells (Supplementary Figure S6A). Both the number of branch points and tube length were increased in the HUVECs after treatment with CM from PA-Huh7 cells and this enhancement was significantly abrogated by additional treatment with *RUNX1* siRNA (Supplementary Figure S6A–C). Our data demonstrate that RUNX1, induced by VEGF released from PA treated hepatoma cells, is one of the key responsible factors enhancing angiogenic activity in ECs.

## 4. Discussion

In the current study, we report increased expression of RUNX1 in liver NPCs, presumably among others in ECs of NASH livers. We describe a novel angiogenic and inflammatory role of RUNX1 in NASH pathogenesis. Signals such as VEGF from PA-treated hepatocytes induce/increase the expression of *RUNX1* in ECs, resulting in an enhanced expression of angiogenic and adhesion molecules in these cells, potentially augmenting increased leucocyte migration and adhesion.

Oxidative stress and inflammation-driven pathological angiogenesis is an important mechanism in the progression of NAFLD from steatosis to NASH to cirrhosis and subsequently HCC [17]. Studies have documented both increased and decreased hepatic expression of the angiogenic genes, such as VEGFA and their cognate receptors in patients with NAFLD when compared to control tissues [5,18]. In our study subjects, we did not observe a significant difference in the gene expression of known angiogenic factors such as *VEGFA, VEGFR1* and *VEGFR2*. However, we found a group of angiogenesis associated genes to be differentially expressed in NASH. Among the transcription factors known to control these DEGs, we became particularly interested in RUNX1, which was significantly positively correlated with the histopathological features of NASH. *PPARγ* was another transcription factor, which was a part of our validated DEGs and showed a good correlation with NAS, steatosis and inflammation degree of the patients and the role of PPARγ in NAFLD is also well established [19].

RUNX1 is a salient factor that is known to control diversification between hematopoietic and endothelial cell lineages [20,21]. Gain and loss of function in RUNX1 has been correlated with cancer progression and metastasis, most notably in acute myeloid leukemia [22]. The role of RUNX1 in enhancing TLR4-mediated inflammation has been demonstrated previously [23]. In our study, there was an increased expression of RUNX1 in liver endothelial cells of NASH patients that significantly correlated with severity of disease, hypothesizing a pathogenic role of endothelial-specific expression of RUNX1 in NASH. Our findings are in concordance with those of Lam et al. who have also identified *RUNX1* as a gene upregulated in CD31-positive vascular ECs obtained from human proliferative diabetic retinopathy fibrovascular membranes [24]. Although RUNX1 was not present in parenchymal liver cells, hepatocytes and cholangiocytes, we did find faint *RUNX1* mRNA expression in Huh7 cells, which is in concordance with the observation of a low RUNX1 nuclear expression in other hepatoma cells such as human Hep3B and mouse AML12 [25]. Furthermore, RUNX1 was shown to regulate TIMP1 (Tissue Inhibitor of Metalloproteinase 1) expression in hepatic stellate cells (HSCs) and to play a role in activating HSCs in a mouse NASH model [14,26]. This may be because in earlier studies, they solely analyzed *RUNX1* in the HSCs and in our study, we focused on endothelial-specific expression of RUNX1 and its potential role in NAFLD pathogenesis. However, it is highly possible that an increased RUNX1 expression in HSCs or other NPCs such as Kupffer cells also contributes to NASH pathogenesis via distinct mechanisms.

Performing in vitro cultures, we observed an increased expression of *RUNX1* in HUVECs which were treated with CM from PA treated hepatoma cells, indicating that non-saturated fatty acids and/or high fat conditions, which are associated with oxidative stress and generation of reactive oxygen species (ROS), may be inducing *RUNX1* expression in HUVECs. Interestingly, a study has reported an increase in mRNA expression of *RUNX1* in liver tissue of a NAFLD guinea pig model, suggesting a regulatory role of *RUNX1* for organic cation transporter N1 (OCTN1) [27]. OCTN1 specifically transports ergothioneine, a natural radical scavenger and therefore, augments its anti-oxidative and anti-inflammatory properties. Previous studies in model organisms have shown that high glucose levels act as a trigger for *RUNX1* expression via ROS–mediated upregulation of hypoxia-inducible factor 1 [24]. Therefore, *RUNX1* expression seems to be triggered by cellular oxidative stress, dietary factors and with regard to hepatic steatosis, this is mediated by VEGF and TGF-β, which were shown to be released from hepatoma cells after PA treatment. The activation of *RUNX1* by TGF-β and other transcription factors such as *SMAD1* has also been well reported in previous studies [28,29].

Overexpression of RUNX1 in ECs resulted in substantial increase in the expression of adhesion molecules VCAM1 and PECAM1 and also that of CCL2, independent of additional treatment with VEGF. Based on this finding, we propose a novel role of RUNX1 in the potential recruitment of inflammatory cells in NASH, because VEGF is known to aggravate endothelial cell chemokine production in vitro and in vivo and functions in the recruitment of monocytes and T cells [16]. Hence, both VEGF and RUNX1 may act together as crucial angiogenic and pro-inflammatory inducers in NASH. We confirmed the angiogenic properties of RUNX1 in ECs and show that RUNX1 may be an important downstream effector of VEGF in mediating endothelial angiogenesis. However, angiogenic factors other than VEGF may also be involved in RUNX1 regulation and need further characterization.

RUNX1 attenuation also led to a decrease in the expression of known RUNX1 target genes, including *CCL2, PI3KCA, PRKCE* and *eNOS*. Furthermore, a decrease in angiogenic activity of ECs while *RUNX1* mRNA expression is silenced validated its role as an angiogenic modulator. The contribution of *RUNX1* towards angiogenesis in steatosis may also be deduced by the fact that many of its target genes, including *eNOS* and *PI3KCA*, are known to be involved in various aspects of angiogenesis, including EC proliferation, sprouting and vascular stabilization during hepatic steatosis and inflammation in high fat diet mice [30,31]. PRKCE is already known to play a critical role in mediating fat-induced hepatic insulin resistance through the buildup of diacylglycerol in NAFLD [32,33,34]. CCL2 is mainly secreted by infiltrating inflammatory monocytes and HSCs in an injured liver and is known to provide pro-angiogenic signals during chronic liver injury [35]. In our study, CCL2 significantly correlated with the severity of human NASH and CCL2 release from ECs was significantly altered by RUNX1 expression, adding novel insight into how RUNX1/CCL2 mediates infiltration of inflammatory cells and angiogenesis in NASH. In addition, the expression of *VEGFR1* was found to be attenuated in HUVECs after *RUNX1* mRNA silencing, implicating that *VEGFR1* may be a vital factor downstream of RUNX1-mediated angiogenesis and disease progression in NAFLD [36]. However, whether *VEGFR1* is a direct target of RUNX1 remains to be determined.

## 5. Conclusions

To summarize, this study implicates the role of *RUNX1* in aberrant liver angiogenesis and inflammation in NASH, by modulating the expression of adhesion molecules and angiogenic properties of ECs. The study not only identifies *RUNX1* as a novel prospective therapeutic target and a biomarker in NASH but also opens opportunities for exploration of the mechanisms of angiogenesis and inflammation in NASH. In vivo *RUNX1* depletion studies that would provide insight into the precise contribution of *RUNX1* towards inflammation and fibrosis in NASH are highly requisite.

## Figures and Tables

**Table 1:** Analysis of mRNA expression of genes associated with angiogenesis in resected hepatic tissue samples of patients with normal liver (N), steatosis (S) and non-alcoholic steatohepatitis (NASH). Data are shown as means ± SD and statistical differences were analyzed by pairwise comparison using a Kruskal–Wallis Test.

	Relative mRNA Expression			*p*-Value		
Gene	N (*n* = 33)	S (*n* = 46)	NASH (*n* = 43)	N/S	N/SH	S/SH
*CCL2*	1.82 ± 1.47	2.30 ± 1.63	3.74 ± 2.13	0.534	0.000	0.002
*CXCL8 (IL8)*	4.33 ± 4.5	7.16 ± 8.99	16.97 ± 17.18	0.404	0.000	0.002
*CXCR4*	1.57 ± 1.18	2.63 ± 1.70	2.87 ± 1.84	0.008	0.002	1.000
*EREG*	2.87 ± 2.26	5.34 ± 4.86	7.48 ± 6.19	0.197	0.001	0.285
*FASN*	3.27 ± 2.07	6.35 ± 5.75	5.67 ± 5.27	0.048	0.077	1.000
*HOMX1 (HO1)*	0.71 ± 0.36	0.67 ± 0.39	0.95 ± 0.66	1.000	0.126	0.024
*NOS3 (eNOS)*	0.72 ± 0.32	0.82 ± 0.52	1.12 ± 0.74	1.000	0.011	0.040
*PIK3CA*	0.61 ± 0.21	0.98 ± 1.22	0.81 ± 0.25	0.013	0.001	1.000
*PPARγ*	0.61 ± 0.24	0.73 ± 0.34	0.97 ± 0.47	0.552	0.001	0.034
*PRKCE*	0.71 ± 0.34	0.96 ± 0.42	1.00 ± 0.60	0.004	0.014	1.000
*PROK2*	2.55 ± 2.17	3.66 ± 4.13	6.49 ± 7.96	1.000	0.058	0.182
*RUNX1*	1.75 ± 1.21	1.90 ± 1.56	3.38 ± 2.55	1.000	0.000	0.002

## Supplementary Materials

The following are available online at https://www.mdpi.com/2073-4409/8/10/1277/s1, Supplementary methods, **Figure S1**: Correlation of validated DEGs with histopathological parameters, **Figure S2**: Representative immunohistochemical images, **Figure S3**: *RUNX1* mRNA expression in endothelial cells maintained in conditioned medium (CM) from palmitic acid (PA) treated hepatoma (Huh7) cells or primary hepatocytes, **Figure S4**: Expression of *RUNX1* in endothelial cells, **Figure S5**: RUNX1 alters expression of adhesion molecules in endothelial cells, **Figure S6**: RUNX1 enhances angiogenic activitiy of endothelial cells, **Table S1**: Age, BMI, steatosis, inflammation, and fibrosis scores of the cohort studied for gene expression analysis, **Table S2**: Age, BMI, steatosis, inflammation, and fibrosis scores of the cohort studied for Immunohistochemical analysis, **Table S3A**: List of genes and assay numbers (Thermo Fisher) used for Fluidigm qRT-PCR, **Table S3B**: List of genes and primers used for qRT-PCR (SYBR Green-Based), **Table S4**: Antibodies used for Immunohistochemistry (IHC) and Flow Cytometry (FC) analysis, **Table S5**: DEGs obtained from mRNA micro-array analysis and associated with GO term angiogenesis are presented with their log2 fold change (FC) and p-values for comparison steatosis (S; *n* = 7) to normal liver (N; *n* = 7), NASH (SH; *n* = 7) to normal liver and NASH to steatosis, **Table S6**: Analysis of mRNA expression of genes associated with angiogenesis in tissue samples of patients with liver steatosis (S), NASH (SH) and normal liver (N) by qRT-PCR. Shown are genes, which are not significantly differentially expressed between the groups.
