# Supplementary material for: Increased Expression of RUNX1 in Liver Correlates with NASH Activity Score in Patients with Non-Alcoholic Steatohepatitis (NASH)
Source: Cells. 2019 Oct 18;8(10):1277. doi: 10.3390/cells8101277 (PMC6830073; doi:10.3390/cells8101277)
Supplement: Supplementary file 1 [file cells-08-01277-s001.pdf]

Supplementary Methods

Microarray studies

Briefly, total RNA was isolated from human liver tissues (normal liver, n = 7, steatosis, n = 7, NASH, n = 7) using RNeasy kit including on column genomic DNA digestion with RNase-free DNase Set (Quiagen, Hilden, Germany). In a pilot study a mRNA microarray experiment was performed using Affymetrix HT-HGU 133 Plus-PM peg plates according to the manufacturer's instructions (Affymetrix Inc., Santa Clara, CA, USA). Sample processing and microarray hybridization were carried out at an Affymetrix Service Provider and Core Facility (Regensburg, Germany, www.kfb-regensburg.de). Pre-processing of the raw data was performed using robust multi-array average, which includes background correction, normalization and summarization [1]. Differentially expressed genes (DEGs) were identified with respect to the diagnosed stage of NAFLD i.e. pairwise comparison of groups. DEGs were identified using R-package LIMMA [2] with a false discovery rate (FDR)-adjusted level of 0.1 and LIMMA calculated log2 fold change (FC) with a p-value <0.05. To identify over-represented GO-categories from the list of DEGs a gene enrichment analysis was performed using GOstats [3]. Data from the micro array experiment are presented in Table S5.

Taqman quantitative Real time PCRs (qRT-PCRs)

Total RNA was isolated from a lager cohort of human liver tissues (normal liver, n = 33, steatosis, n = 46, NASH, n = 43; see also Table S1) using RNeasy kit including on column genomic DNA digestion with RNase-free DNase Set (Quiagen, Hilden, Germany). For quantitative RT-PCR analysis, we used the Fluidigm's BioMark high-throughput quantitative (q) PCR chip platform (Fluidigm Corporation, San Francisco, CA, USA) with pre-designed gene expression assays from ThermoFisher (suppl. Table S3A) according to the manufacturer's instructions [4]. The data were analyzed using the ΔΔCt method [5] and the expression values were normalized to the expression levels of the housekeeping genes (*HPRT, YWHAZ*) and shown in Table 1 and Table S6.

SYBR Green-based qRT-PCR Assays

Briefly, total RNA from trypsinized cells (in vitro culture experiments) was isolated with Trizol reagent (Invitrogen, Carlsbad, CA, USA) following the manufacturer's instructions. RNA quantified at 260/280 nm with ThermoScientific Nanodrop 2000 Spectrophotometer. The absorption ratio A260 nm/A280 nm between 1.90 and 2 was taken into consideration for cDNA preparation. First strand cDNA was synthesized from 1µg of total RNA with reverse transcriptase (Thermo Scientific Verso cDNA synthesis kit) according to manufacturer instructions. Quantitative real time PCR was carried using SYBR green PCR master mix (Applied Biosystems, Foster City, CA, USA) carried out in real time PCR machine. Dissociation curve was generated at the end of each PCR to verify that a single DNA species was amplified. The following cycling parameters were used: start at 95 °C for 5 min, denaturing at 95 °C for 30 s, annealing at 60 °C for 30 s, elongation at 72 °C for 30 s, and a final 5 min extra extension at the end of the reaction to ensure that all amplicons were completely extended and repeated for 40 amplification cycles. The data were analyzed using the ΔΔCt method [5] and the expression values were normalized to the expression levels of the housekeeping gene, *18S* RNA.

Transcription factor Analysis

The angiogenic DEGs validated in RT-PCR studies were further dissected bio-informatically to study the transcription factors controlling them in all the subject groups (normal, steatosis and steatohepatitis) using DAVID 6.8 and JASPAR.

Immunohistochemistry studies

From all tissue samples, human liver tissue sections were fixed in 10% formalin and embedded in paraffin. They were cut with a sliding microtome into 2.5 μm sections for histology. Liver sections were deparaffinized and rehydrated. Thereafter, endogenous peroxidase was blocked for 20min with 3% hydrogen peroxidase in methanol. Antigen retrieval was done in Tris-EDTA buffer (pH: 9) for 6 min at boiling temperature. After washing under running water and giving a protein block with BSA for 1 h, they were incubated overnight at room temperature with anti RUNX1 primary antibodies (list of antibodies used given in Table S4). Thereafter, the specimen was incubated with the PolyExcel Target Binder for 10 min followed by a PolyExcel HRP labeled polymer using recommended 10 min incubation (PathNSitu Biotechnologies, Hyderabad, India). Staining was completed by a 5–10 min incubation with 3, 3′-diaminobenzidine (DAB) substrate-chromogen which resulted in a brown-colored precipitate at the antigen site. Counterstaining was done using hematoxylin. Immunohistochemical (IHC) scoring was done on a scale of 1-4 by counting RUNX1 positive brown cells per field. Four to five different fields were randomly selected for each sample and mean was taken. Internal negative antibody controls comprised of staining the NASH samples with RUNX1 antibody but without secondary antibody. These sections were counter-stained with hematoxylin.

Isolation of mouse primary hepatocytes and liver sinusoidal endothelial cells

Primary liver cells were isolated from control C57BL6 mice for *in vitro* studies. Primary hepatocytes (PH) and liver sinusoidal endothelial cells (LSECs) were isolated as described elsewhere with minor modifications [6]. Briefly, mice were intraperitoneally anesthetized with a combination of 100mg/kg ketamine and 5mg/kg midazolam. A mid-abdominal incision towards the sternum was made and the intestines were displaced to expose the portal vein. Liver was perfused (27G catheter) through the portal vein with pre-warmed Buffer 1 (HEPES with EGTA) for 10 min at a flow rate of 20 mL/min. Simultaneously the cava vein was cut to allow outflow of the solution. After perfusion, the liver was digested with pre- warmed Buffer 2 (HEPES with 0.015% collagenase) for 30min at a flow rate of 5mL/min. The resultant digested liver was excised, cut up and in vitro digestion was performed with Buffer 2. Disaggregated tissue was filtered using a 100μm nylon strainer and filtrate was centrifuged at 50× *g* for 5 min at 4^0C. The pellet contained hepatocytes and supernatants contained the non-parenchymal cells. For isolation of LSECs, the supernatant was centrifuged at 800g for 10min at 4^0C, and the obtained pellet was resuspended in PBS and centrifuged at 800g for 20min through a 25–50% Percoll gradient (Sigma–Aldrich, New Delhi, India) at room temperature. The interface of the gradient containing Kupffer cells and LSEC was pre-cultured on 37-mm tissue culture plates for 25min at 37^0C. The non-attached cells were enriched in LSECs.

Preparation of steatotic liver cells and BODIPY staining

For the preparation of steatotic hepatocytes/hepatoma cells, primary hepatocytes (PH) or Huh7 cells were grown in Dulbecco's modified Eagle's medium (DMEM) supplemented with 10% fetal bovine serum (Himedia Laboratories, Mumbai, India), penicillin (400U/ml), streptomycin (50μg/ml), L-glutamine (300μg/ml). Free fatty acid treatment of cells was done mainly according to previously published methods, where PH or Huh7 cells at 80% confluency were exposed to Palmitic acid-BSA (Himedia Laboratories, Mumbai, India) for 48 h [7]. Briefly, Palmitic acid (51.2mg) was dissolved in 100% ethanol (1ml) to make 200mM of stock solution. About 10% FFA low-endotoxin BSA was prepared in DMEM. 4mM palmitate-BSA conjugate in 10% BSA or 10% BSA was diluted 20× in complete medium containing 10% FBS to prepare 0.2mM or 200μM palmitate working treatment or BSA control medium. The total BSA concentrations in control and palmitate containing medium were kept the same to avoid differential protein binding effect on compounds. BSA-treated cells served as controls. After 48h, conditioned media (CM) was collected from BSA-treated and palmitate-BSA (PA) treated cells, sterile filtered (0.45 μm pore size membrane filter), and stored in aliquots at −20 °C until further use.

Fat deposition in PH and Huh7 cells were studied by BODIPY staining. Cells were washed twice with PBS before being fixed with 10% formaldehyde in PBS for 15min. After two washes in PBS, cells were then stained for 15-20min in BODIPY stain (1μg/ml, Thermofisher Scientific, Waltham, MA, USA). Representative micrographs were captured at 40× magnification using a fluorescence microscope.

Maintenance of endothelial cells in culture medium from palmitic acid treated Huh7 cells

HUVECs (Thermo Fisher Scientific, Waltham, MA, USA) or mouse LSECS were plated on 0.2% gelatin or collagen coated plates, respectively. Both endothelial cells were maintained in Endothelial Cell Basal Medium (Lonza, Mumbai, India) 10% FBS, 1% antibiotics (penicillin and streptomycin, Himedia Laboratories, Mumbai, India) and growth factors (endothelial cell growth medium supplement, Lonza, Mumbai, India). Cells were cultured at 37°C in a 5% CO_2 humidified environment. HUVECs were used between passages 2 and 4. LSECs or HUVECs were treated with CM from BSA and PA-treated hepatocytes or Huh7 cells, respectively, for 24 h and then assayed for *RUNX1* gene expression. HUVECs were also treated directly with BSA and 200μM PA-BSA for 24h and then studied for the gene expression of *RUNX1*.

Matrigel assays

For angiogenesis assays, serum-starved HUVECs (50,000 cells per well) were plated on 200μL of 5mg/mL polymerized matrigel onto 24 well plates, and then treated as described. In all conditions, after 18-20h, each well of HUVECs was analyzed under a phase contrast microscope with 4x objective [6]. Tubules in each field were imaged and branch points and tube length from 3-5 random fields in each well were counted.

ELISA assays for VEGF, PDGF-BB, TGF-β and CCL2

The levels of VEGF, PDGF-BB, TGF- β and CCL2 were analyzed in culture medium of untreated, BSA-treated and PA-treated Huh7 cells by ELISA using Thermofisher Scientific ELISA kits as per manufacturer's instructions (ThermoFisher Scientific, Waltham, MA, USA; Invitrogen, Carlsbad, CA, USA). Standard curve was drawn using standards provided in the kit and each analyte concentration was calculated from the standard curve. The assays were normalized to 10^6 cells per ml.

Supplementary Tables

Table 1. Age, BMI, steatosis, inflammation- and fibrosis scores of the cohort studied for gene expression analysis. Data are shown as median and range of values.

	Normal liver	Steatosis	NASH
Patients (male/female)	33 (16/17)	46 (22/24)	43 (25/18)
Age [y]	58 (20-82)	60 (24-84)	65 (33-82)
BMI [kg/m^2]	25 (18-30)	28 (22-46)	28 (21-58)
Steatosis [a]	0	2 (1-3)	2 (1-3)
Inflammation [b]	0	0 (0-2)	2 (1-3)
Fibrosis [c]	0	0 (0-4)	2 (1-3)
Ballooning [d]	0	0 (0-1)	2 (1-2)
NASH activity score [e]	0 (0)	1 (1-2)	3 (3-4)

Human liver tissues for mRNA expression analysis were histologically examined for patients without NAFLD, patients with simple liver steatosis and patients with NASH as described earlier [8]. [a] Steatosis was scored as <5% steatosis (0), 5 to 33% steatosis (1), >33 to 66% steatosis (2) and >66% steatosis (3). [b] Inflammation was scored as no foci / 20 × field (0), <2 foci / 20 × field (1), 2–4 foci /20×field (2) and >4 foci / 20 × field (3). [c] Fibrosis was defined as no fibrosis (0), zone 3 perisinusoidal/pericellular fibrosis; focally or extensively present (1), zone 3 perisinusoidal/ pericellular fibrosis with focal or extensive periportal fibrosis (2), zone 3 perisinusoidal/pericellular fibrosis and portal fibrosis with focal or extensive bridging fibrosis (3) and cirrhosis (4). [d] Ballooning was scored by the number of enlarged hepatocytes as none/field (0), few ballooned hepatocytes/field (1), many ballooned hepatocytes/field (2). [e] NASH activity was calculated as described elsewhere (8)

and NASH activity (from 0 to 8) were assigned to NASH activity scores (NAS) as following: 0, normal; 1-2 = 1, no NASH; 3-4 = 2, no NASH; 5-6 = 3, mild NASH and 7-8 = 4, severe NASH. Patients with a score 3 or 4 were designed as suffering from NASH.

Table 2. Age, BMI, steatosis, inflammation- and fibrosis scores of the cohort studied for Immunohistochemical analysis Data are shown as median and range of values.

	Normal liver	NASH
Patients (male/female)	15 (12/3)	21 (16/5)
Age [y]	46 (22-62)	44 (28-68)
BMI [kg/m^2]	23.5 (18-28)	28 (21-42)
Steatosis [a]	0	2 (1-3)
Inflammation [b]	0	2 (1-4)
Fibrosis [c]	0	2 (0-4)
Ballooning [d]	0	1 (1-2)
NASH activity score [e]	0	3 (2-4)

For scoring and definition of steatosis, inflammation, fibrosis, ballooning and NASH activity score see supplementary table S1.

Table 3A. List of genes and assay numbers (Thermo Fisher) used for Fluidigm qRT-PCR.

Gene	Assay No.	Gene	Assay No.
CCL2	Hs00234140_m1	SIRT1	Hs01009005_m1
IL8 (CXCL8)	Hs00174103_m1	CDKN1B (p27)	Hs01597588_m1
CXCR4	Hs00607978_s1	FLT1 (VEGFR1)	Hs01052961_m1
EREG	Hs00914313_m1	HOXB3	Hs01587922_m1
FASN	Hs01005622_m1	KDR (VEGFR2)	Hs00911700_m1
HMOX1 (HO1)	Hs00157965_m1	LRP5	Hs00182031_m1
NOS3 (eNOS)	Hs01574659_m1	MEIS1	Hs01017441_m1
PIK3CA	Hs00907957_m1	PPARA/PPARα_total	Hs00231882_m1
PPARG	Hs01115513_m1	VCAM1	Hs01003372_m1
PRKCE	Hs00178455_m1	VEGFA	Hs00900055_m1
PROK2	Hs01587689_m1	HPRT1	Hs02800695_m1
RUNX1	Hs01021970_m1	YWHAZ	Hs03044281_g1

Table 3B. List of genes and primers used for qRT-PCR (SYBR Green-Based).

Gene	Forward	Reverse
CXCR4	TGGCTGAAAAGGTGGTCTATG	TGATGTGCTGAAACTGGAACA
CCL2	TCCCAAAGAAGCTGTGATCTTCA	TCTGGGGAAAGCTAGGGGAA
CXCL8	ACTGAGAGTGATTGAGAGTGGAC	ACAACCCTCTGCACCCAGTT
RUNX1	ACTCGGCTGAGCTGAGAAATG	GACTTGCGGTGGGTTTGTG
PRKCE	GAGCCAGAAGGAAGAGTGTATG	GGGACCTTGTAGTTGTGGATAC
PPARγ	CCAGGTTTGCTGAATGTGAA	TGTCTGTCTCCGTCTTCTTGA
VEGFR1	ATGGTCTTTGCCTGAAATGG	AGCCAGTGTGGTTTGCTTGA
VEGFR2	TGTATGTCCCACCCCAGATT	CTCTTCCTCCAACTGCCAAT
VCAM1	AGTTGAAGGATGCGGGAGT	GCAAAATAGAGCACGAGA
eNOS (NOS3)	GCATCACCAGGAAGAAGACC	GGAGCCATACAGGATTGTCG
PI3KCA	CGCATTTCCACAGCTACACC	AGCCATTCATTCCACCTGGG
ICAM1	GGCTGGAGCTGTTTGAGAAC	ACTGTGGGGTTCAACCTCTG
PECAM1/CD31	CCAGTGTCCCCAGAAGCAAA	TGATAACCACTGCAATAAGTCCTTTC

Table 4. Antibodies used for Immunohistochemistry (IHC) and Flow Cytometry (FC) analysis.

Antibody	Cat. No.	Clonality / Host	Supplier	Fluorochrome	Dilution
Primary antibody					
RUNX1 (IHC)	sc-365644	Mouse	Santa Cruz	Unconjugated	1/100
PECAM1/CD31 (FC)	550389	Mouse	BD Biosciences	APC	1/100
VCAM1 (FC)	551148	Mouse	BD Biosciences	PE-Cy5	1/100
VEGF-A (Blocking)	E-AB-34220	Rabbit	Elabsciences	Unconjugated	1/1000

Table 5. DEGs obtained from mRNA micro-array analysis and associated with GO term angiogenesis are presented with their log2 fold change (FC) and p-values for comparison steatosis (S; n=7) to normal liver (N; n=7), NASH (SH; n=7) to normal liver and NASH to steatosis.

	**Chip/FC**		**Chip/FC**		**Chip/FC**	
Gene symbol	S/N	P-value	SH/N	P-value	SH/S	P-value
CCL2 [a]	0.205	0.925	1.610	**0.017**	1.405	**0.039**
CDKN1B (p27)[a]	-0.148	0.229	-0.525	**0.000**	-0.377	**0.001**
CXCL8 (IL8)	0.437	0.880	2.766	**0.014**	2.329	**0.040**
EREG	0.122	0.671	0.510	**0.004**	0.388	**0.027**
FASN [b]	0.460	**0.007**	0.312	0.065	-0.148	0.500
HMOX1 (HO1) [a]	-0.841	**0.004**	0.010	0.999	0.851	**0.003**
HOXB3	-0.070	0.883	-0.455	**0.013**	-0.384	**0.037**
LRP5 [b]	-0.436	**0.014**	-0.346	**0.046**	0.090	0.783
MEIS1	0.274	0.190	-0.324	0.092	-0.599	**0.002**
NOS3 (eNOS)	-0.093	0.502	0.177	0.090	0.270	**0.008**
PIK3CA [b]	-0.196	0.066	-0.321	**0.002**	-0.125	0.275
PPARA [a,b]	-0.277	0.175	-0.683	**0.000**	-0.405	**0.028**
PRKCE [a,b]	0.174	0.771	0.818	**0.009**	0.643	**0.041**
PROK2	0.653	0.504	2.011	**0.005**	1.358	0.061
RUNX1	0.021	0.919	0.197	**0.003**	0.176	**0.007**
SIRT1 [a,b]	-0.237	0.345	-0.640	**0.002**	-0.403	0.052
VCAM1 [a]	-0.818	**0.001**	-0.914	**0.000**	-0.096	0.870

[a] associated with GO-term hypoxia; [b] associated with GO-term lipid metabolism.

Table 6. Analysis of mRNA expression of genes associated with angiogenesis in tissue samples of patients with liver steatosis (S), NASH (SH) and normal liver (N) by qRT-PCR. Shown are genes, which are not significantly differentially expressed between the groups. Data are shown as means ± SD and statistical difference were analyzed by pairwise comparison using Kruskal-Wallis Test.

	**relative mRNA expression**						**P-value**		
**Gene**	**N (n=33)**		**S (n=46)**		**SH (n=43)**		**N/S**	**N/SH**	**S/SH**
CDKN1B (p27)	0.57	± 0.20	0.89	± 0.89	0.60	± 0.25	0.052	1.000	0.053
Flt1 (VEGFR1)	0.74	± 0.34	0.79	± 0.33	0.95	± 0.53	1.000.	0.088	0.220
HoxB3	2.05	± 2.48	3.04	± 3.99	1.65	± 1.70	0.481	1.000.	0.099
KDR (VEGFR2)	0.47	± 0.21	0.52	± 0.26	0.54	± 0.22	0.907	0.509	1.000.
LRP5	1.07	± 0.77	1.11	± 1.14	1.32	± 1.04	1.000.	0.857	1.000.
MEIS1	0.64	± 0.27	0.72	± 0.35	0.63	± 0.34	0.983	1.000.	0.596
PPARα	0.73	± 0.28	0.81	± 0.30	0.80	± 0.31	0.798	0.957	1.000.
SIRT1	0.75	± 0.25	0.84	± 0.36	0.89	± 0.28	0.641	0.178	1.000
VCAM-1	0.68	± 0.30	0.74	± 0.35	0.76	± 0.30	1.000.	0.866	1.000.
VEGFA	1.07	± 0.44	1.24	± 0.99	1.12	± 0.58	0.857	1.000.	1.000

Supplementary References

1. Irizarry RA, Hobbs B, Collin F, Beazer-Barclay YD, Antonellis KJ, Scherf U, et al. Exploration, normalization, and summaries of high density oligonucleotide array probe level data. Biostatistics 2003, 4, 249–264.
2. Smyth GK. Linear models and empirical bayes methods for assessing di_erential expression in microarray experiments. Stat Appl Genet Mol Biol 2004, 3, 3.
3. Falcon S, Gentleman R. Using go stats to test gene lists for go term association. Bioinformatics 2007, 23, 257–258.
4. Spurgeon SL, Jones RC, Ramakrishnan R. High throughput gene expression measurement with real time PCR in a microfluidic dynamic array. PLoS One 2008, 3, e1662.
5. Livak KJ, Schmittgen TD. Analysis of relative gene expression data using real-time quantitative PCR and the 2(-Delta Delta C(T)) Method. Methods 2001, 25, 402–408.

6. Kaur S, Tripathi D, Dongre K, Garg V, Rooge S, Mukopadhyay A, et al. Increased number and function of endothelial progenitor cells stimulate angiogenesis by resident liver sinusoidal endothelial cells (SECs) in cirrhosis through paracrine factors. J Hepatol 2012, 57, 1193–1198.
7. Luo Y, Rana P, Will Y. Palmitate increases the susceptibility of cells to drug-induced toxicity: an in vitro method to identify drugs with potential contraindications in patients with metabolic disease. Toxicol Sci 2012, 129, 346–362.
8. Weiss TS, Lupke M, Ibrahim S, Buechler C, Lorenz J, Ruemmele P, et al. Attenuated lipotoxicity and apoptosis is linked to exogenous and endogenous augmenter of liver regeneration by different pathways. PLoS One 2017, 12, e0184282.
9. Kleiner DE, Brunt EM, Van Natta M, Behling C, Contos MJ, Cummings OW, et al. Nonalcoholic Steatohepatitis Clinical Research Network. Design and validation of a histological scoring system for nonalcoholic fatty liver disease. Hepatology 2005, 41, 1313–1321.

Supplementary Figures

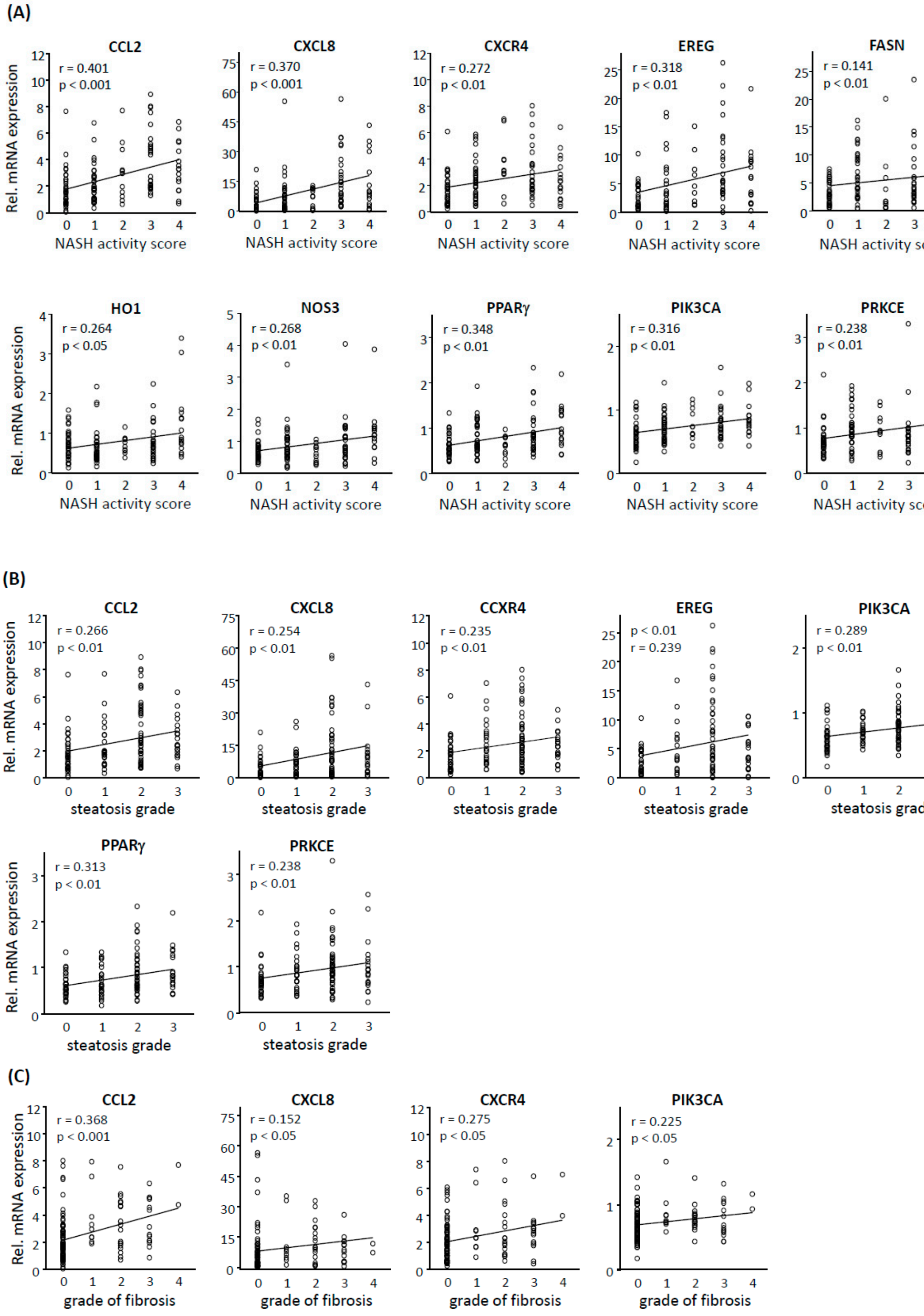

Fig. S1

Figure 1. Correlation of validated DEGs with histopathological parameters. Expression of mRNA was analyzed by qRT-PCR in liver tissue samples from patients with NASH (n = 43), hepatic steatosis (n = 46) and normal liver tissue (n = 33) and correlated to histopathologic proven (A) NASH activity score (B) steatosis grade (C) fibrosis grade and (D) inflammation grade. *HPRT* mRNA expression was determined for normalization, statistical differences between several grades were analyzed by Kruskal-Wallis Test ($p < 0.05$ was considered significant) and 'r' denotes Pearson's correlation coefficient.

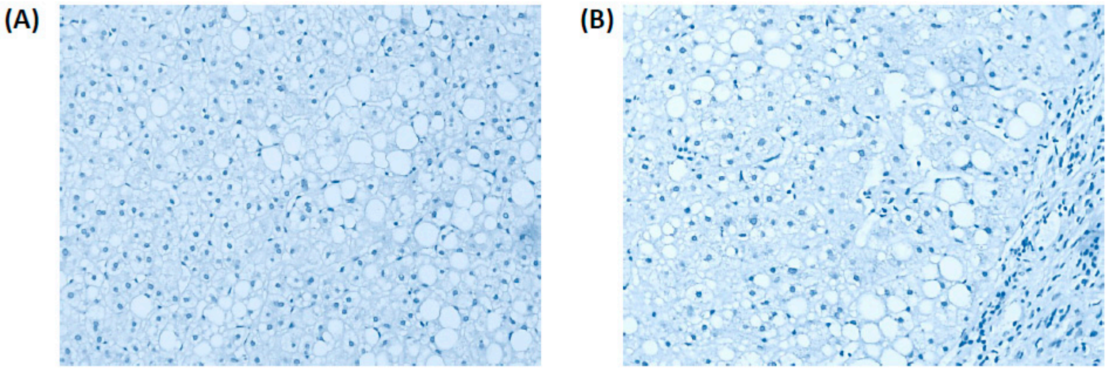

Figure 2. Representative immunohistochemical images showing negative antibody controls (only primary antibody without secondary antibody) in patients (20x) (A) NASH patient with NAS = 6, Fibrosis = 1 and (B) NASH patient with NAS = 6, Fibrosis = 4.

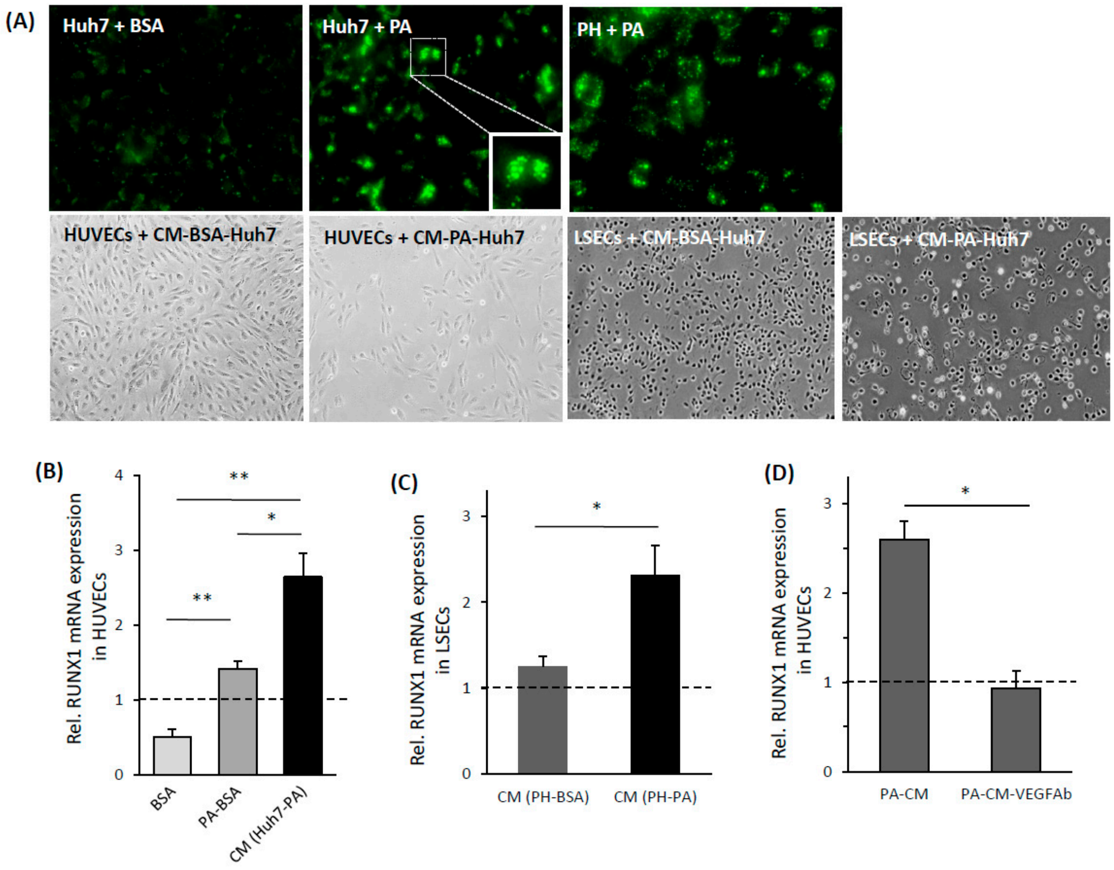

Figure 3. *RUNX1* mRNA expression in endothelial cells maintained in conditioned medium (CM) from palmitic acid (PA) treated hepatoma (Huh7) cells or primary hepatocytes. (A) Florescence images (40x objective) of BODIPY stained Huh7 cells treated with Bovine serum albumin (BSA), Huh7 cells or primary hepatocytes (PH) treated or 0.2 mM Palmitic acid (PA) for 48 hours. Phase contrast images (10x objective) of HUVECs treated with conditioned media (CM) from BSA- or PA-treated Huh7 cells. Phase contrast images (10x objective) of LSECs treated with conditioned media from BSA- or PA-treated primary hepatocytes. (B) Relative *RUNX1* mRNA expression in HUVECs incubated with BSA or palmitate and CM from palmitate treated Huh7 cells (n = 3). Dotted line represents untreated HUVECs (controls) (C) Relative *RUNX1* mRNA expression in LSECs after treatment with CM from BSA or palmitate treated PH (n = 3). Dotted line represents untreated LSECs (controls). (D) Relative *RUNX1* mRNA expression in HUVECs incubated with CM from

palmitate treated Huh7 cells with or without addition of VEGF-A antibody (PA-CM-VEGFAb) (n=3). Dotted line represents HUVECs treated with CM from BSA-Huh7 cells with and without VEGF-A antibody (respective controls). Data represent mean ± SD. * p < 0.05; ** p < 0.001.

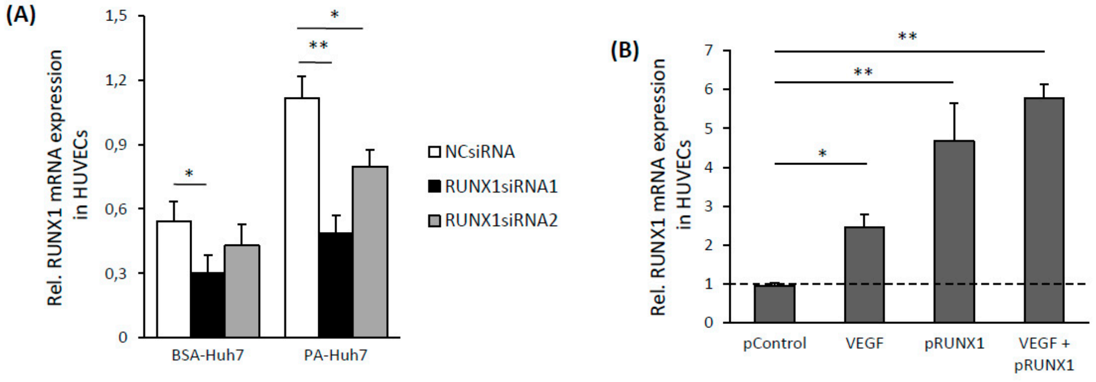

Figure S4: Expression of *RUNX1* in endothelial cells. (A) RNA expression (fold change) of *RUNX1* in HUVECs incubated with CM from BSA-Huh7 and PA-Huh7 and then treated with *RUNX1* siRNAs or negative control (NC) siRNA (n = 3). (B) HUVECs, transfected with RUNX1 expression plasmid (pRUNX1), control plasmid (pControl i.e empty vector) and/or incubated with VEGF (10ng/ml) were analyzed for mRNA expression of *RUNX1*. HUVECs without any treatment were used as respective controls (Dotted line) (n = 3). 18S RNA expression was used for normalization. Data represent mean ± SD. * p < 0.05; ** p < 0.001.

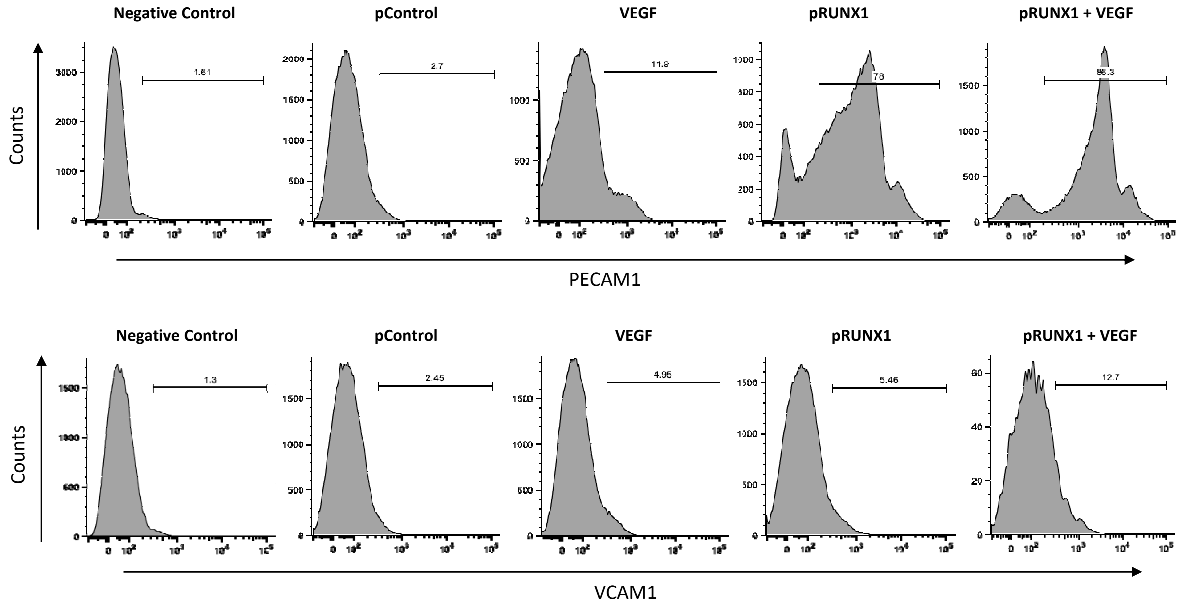

Figure 5. RUNX1 alters expression of adhesion molecules in endothelial cells. Representative histograms by flow cytometry demonstrate induction of PECAM1 and VCAM1 expression in HUVECs transfected with RUNX1 expression plasmid (pRUNX1), control plasmid (pControl, empty vector) and/or incubated with VEGF (10 ng/ml). Endothelial cells alone without any labeled antibodies were used as negative controls.

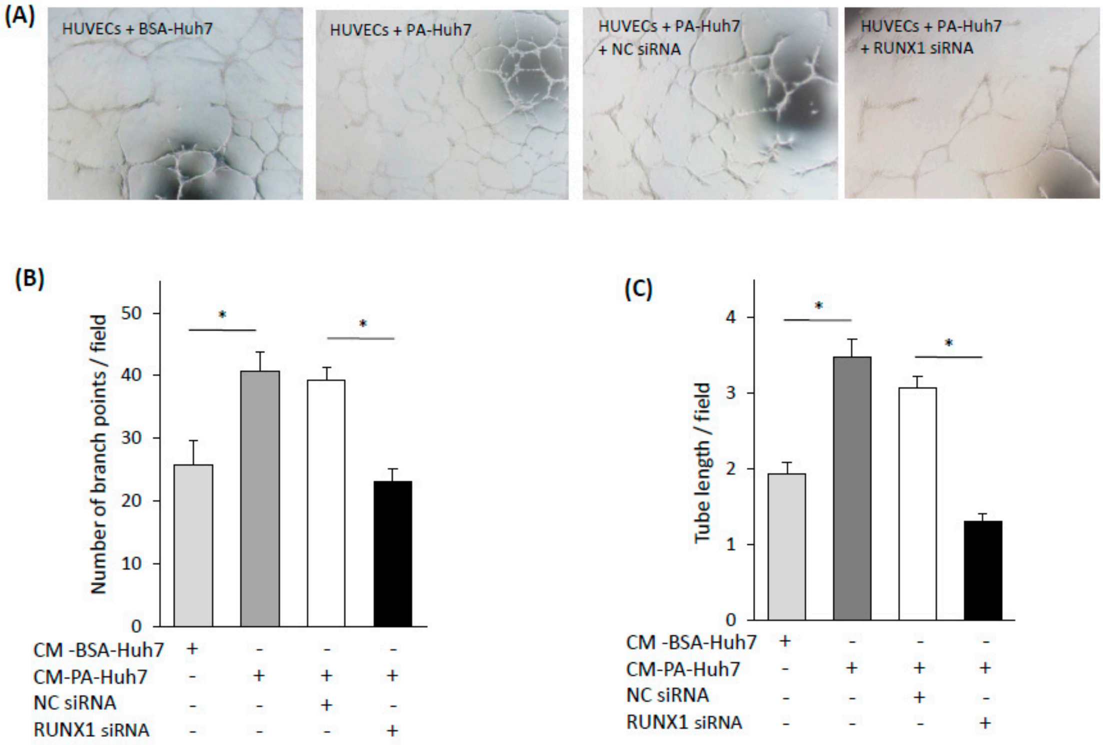

Figure S6. RUNX1 enhances angiogenic activity of endothelial cells. **(**A) Representative tube formation images of HUVECs on matrigel (4x objective) incubated with CM from BSA-Huh7 cells, PA-Huh7 cells, and PA-Huh7 CM treated with *RUNX1* siRNA or NC siRNA. (B) Average number of branch points per field and (C) tube length per field formed by HUVECs on matrigel under conditions described in (A) (n = 3, each). Data represent mean ± SD. * $p < 0.05$.
